# Smoking, Cardiac Symptoms, and an Emergency Care Visit: A Mixed Methods Exploration of Cognitive and Emotional Reactions

**DOI:** 10.1155/2012/935139

**Published:** 2012-09-10

**Authors:** Karyn A. Tappe, Edwin D. Boudreaux, Beth Bock, Erin O'Hea, Brigitte M. Baumann, Steven M. Hollenberg, Bruce Becker, Gretchen B. Chapman

**Affiliations:** ^1^Department of Biostatistics and Epidemiology, Center for Health Behavior Research, University of Pennsylvania, Philadelphia, PA 19104, USA; ^2^Departments of Emergency Medicine, Psychiatry, and Quantitative Health Sciences, University of Massachusetts Medical School, Worcester, MA 01655, USA; ^3^Centers for Behavioral and Preventive Medicine, Providence, RI 02903, USA; ^4^Department of Psychology, Stonehill College, Easton, MA 02357, USA; ^5^Division of Clinical Research, Department of Emergency Medicine, Cooper University Hospital, Camden, NJ 08103, USA; ^6^Division of Cardiovascular Diseases, Cooper University Hospital, Camden, NJ 08103, USA; ^7^Department of Emergency Medicine, Warren Alpert Medical School, Brown University, Providence, RI 02903, USA; ^8^Department of Psychology, Rutgers University, Piscataway, NJ 08854-8097, USA

## Abstract

Emergency departments and hospitals are being urged to implement onsite interventions to promote smoking cessation, yet little is known about the theoretical underpinnings of behavior change after a healthcare visit. This observational pilot study evaluated three factors that may predict smoking cessation after an acute health emergency: perceived illness severity, event-related emotions, and causal attribution. Fifty smokers who presented to a hospital because of suspected cardiac symptoms were interviewed, either in the emergency department (ED) or, for those who were admitted, on the cardiac inpatient units. Their data were analyzed using both qualitative and quantitative methodologies to capture the individual, first-hand experience and to evaluate trends over the illness chronology. Reported perceptions of the event during semistructured interview varied widely and related to the individual's intentions regarding smoking cessation. No significant differences were found between those interviewed in the ED versus the inpatient unit. Although the typical profile was characterized by a peak in perceived illness severity and negative emotions at the time the patient presented in the ED, considerable pattern variation occurred. Our results suggest that future studies of event-related perceptions and emotional reactions should consider using multi-item and multidimensional assessment methods rated serially over the event chronology.

## 1. Introduction

Smoking cessation can reverse some of the adverse health effects associated with smoking, and can prevent new health problems from developing [1, 2]. A health event, like an emergency department (ED) visit, can serve as a “teachable moment” with the potential to motivate patients towards health behavior changes such as smoking cessation. Several lines of research converge to support this contention. Placebo or minimal treatment “control” groups in randomized clinical trials of smokers experiencing a myocardial infarction exhibit long-term cessation rates ranging from 34% [3] to 59% [4], which exceeds those found in most “active treatment” groups in trials of highly motivated, healthy participants (~25–35%) and far exceeds the prolonged cessation rate of community-based, unassisted quitters (about 3–5% per year) [5, 6]. 

These increased cessation and quit attempt rates are seen in emergency departments (EDs) as well. Bock and colleagues (2008), in a randomized controlled trial of individuals treated in the chest pain observation unit of an ED, found that the *control* group, which received no active intervention, demonstrated a 15% continuous quit rate for 3 months after discharge [7]. Boudreaux and colleagues found that 50% of individuals treated in an ED attempted to quit within one month of the visit and 19% made successful change defined as achieving 7-day abstinence [8]. Those with more serious disease or test results may be particularly amenable to smoking cessation [9]. Supporting this concept, those admitted to the hospital through the ED were more likely to successfully quit compared to those discharged directly from the ED [8]. However, it is important to note that despite these increased cessation rates, research shows that even individuals with serious medical problems often return to smoking in the long term, with relapse rates up to 50% [4, 10–13]. 

Despite the importance of the health consequences of smoking and the clinical popularity of the “teachable moment” concept in EDs and other healthcare environments [14, 15], formal examination of the mechanisms that translate a specific health event into early behavior change, and identification of which mechanisms facilitate transition to enduring behavior change, have received remarkably little attention. The present study aims to bridge that research gap by introducing a novel explanatory model for behavior change among tobacco users presenting to the ED with suspected cardiac symptoms. We employed both qualitative and quantitative methods to complete an initial exploration of three key constructs that capture a tobacco-using patient's experience of an acute cardiac health event and that may have potential to impact behavior change: perceived illness severity, event-related emotional reactions, and smoking-related causal attributions. These constructs, outlined below and discussed in depth elsewhere [16], were selected based on their theoretical prominence, empirical support, alignment with the investigators' clinical observations, and potential to inform intervention development.

### 1.1. Perceived Illness Severity

Perceived illness severity is defined as the patient's perception of the seriousness of his or her current health problem. The present usage differs in an important way from past conceptualizations (e.g., Health Belief Model [13, 17]); previous studies have typically examined perceptions of an illness the individual “does not” yet have (future focused), whereas our formulation focuses on an acute health problem the individual “does” have (reality focused). 

It is important to realize that an individual's perception of the seriousness of a current health event will likely change over time, and this variability may have important implications for both measurement and hypothesis generation. For example, an individual may initially perceive chest pain as dyspepsia. If it persists, he may perceive it as more severe and contact his physician or go to the ED. After testing, if confirmed as dyspepsia, the perceived severity will decrease once more. However, if confirmed as a myocardial infarction, perceived severity is likely to remain high. Whether health behavior change is most strongly associated with the peak severity rating, average severity ratings across the event chronology, or some other pattern of ratings across time, is not clear. Garnering richer descriptions of how patients perceive the seriousness of their symptoms over time through the use of qualitative, semistructured interviews in combination with rating scales, is critical to guiding operationalization and measurement of perceived illness severity.

### 1.2. Event-Related Emotional Reactions

 The experience of medical illness, especially acute symptoms warranting emergency attention, can elicit strong emotional reactions, reactions that can influence an individual's behavior, according to self-regulation theory [18, 19]. Research on how such emotional responses can inspire or retard health behavior change has lagged far behind investigations into the traditional cognitive constructs that make up most health behavior theories [19, 20]. Our study explored the valence (positive versus negative), stability, and multidimensionality of the emotional response over the course of the emergency care episode.

### 1.3. Smoking-Related Causal Attribution

 Causal attribution refers to how an individual's behavior is influenced by his understanding of how that behavior is causally related to his illness [18, 19]. In the context of an acute health event, smoking-related causal attribution is defined as the patient's perception that his medical problem is caused or made worse by smoking. While evidence strongly suggests that smokers, including those treated in EDs, who believe their health problems are smoking-related are more likely to quit (e.g., [7, 8, 21]), the literature does not provide much guidance on the nuances of the association. For example, smoking related causal attributions may have different associations with behavior change depending upon its relative importance compared to other perceived causes, like genetic factors, stress, or even other health behaviors, such as diet and exercise. Causal attribution may also moderate the strength of the associations between other constructs, such as perceived severity and emotional reactions, and behavior change. Illnesses perceived as severe may lead to behavior change only when the smoker causally links the illness to smoking; in the absence of such causal attribution, even a life-threatening illness may not inspire behavior change. These interdependencies have not yet been tested empirically, and this study will lay the groundwork for future measurement and model development.

Our study was designed to gather both qualitative and quantitative data on smokers' perceptions of severity, emotions, and causal attributions by patients with suspected cardiac symptoms. The purpose of the qualitative component was to gather a more textured understanding of the first-hand cognitive and affective experiences of the health event as it unfolded over time. Qualitative research is developing a growing role in the understanding of patient experience and, from a practical side, aids in the development of surveys and other quantitative tools that do not yet have a solid theoretical basis. The purpose of the quantitative ratings was to help examine differences in ratings across subjects (i.e., between-subject variability), changes in the ratings over time (i.e., within-subject variability), and the associations between the three constructs. Such quantification is important to help guide decisions about whether measurement across multiple time anchors remains necessary. The results should help to more precisely operationalize our constructs, thereby fostering the creation of standardized measures and theoretical models for use in future studies.

The aims of the present study are as follows:

1. to qualitatively explore the cognitive and affective experience of smokers who experience suspected cardiac symptoms using a novel theoretical model not previously tested,
2. to quantify how the experiences of the participants change over time during the course of a hospital visit and how the timing of interview may impact future survey results,
3. to use the results from the qualitative semistructured interviews to categorize experiences for development of future questionnaire items.

## 2. Materials and Methods

### 2.1. Study Design

This observational study focused on regular tobacco users experiencing cardiac-related symptoms, because cardiac disease commonly results from tobacco use [22], and because cardiac events often prompt tobacco cessation that follows patterns of relapse similar to most quit attempts [23]. This sample, therefore, provides the potential to develop theoretical constructs pertaining to how a health event can inspire both short-and long-term behavior change. Research staff used a semistructured interview to elicit the participant's cognitive and emotional reactions over the course of his or her health event. The study was approved by the Institutional Review Boards of all hospitals where participants were recruited.

### 2.2. Participants

Recruitment for this study took place in three regional tertiary care hospitals: one in Camden, NJ, USA and two in Providence, RI, USA. Our focus was on those patients who had symptoms significant and specific enough to warrant specialized tests for cardiac events—in other words, a sample with high probability of a cardiac event based on their symptomatology. However, within this subset, we also wished to capture a breadth of severity and ensure a heterogeneous sample, and, therefore, two equal groups of patients were recruited at each site. A high-severity group (e.g., those with suspected or confirmed cardiac event) comprised patients who presented to the ED for symptoms such as chest pain, chest pressure, or shortness of breath; who underwent, at a minimum, an electrocardiogram and assessment of cardiac enzyme levels (e.g., troponin), and who were subsequently hospitalized in a cardiac inpatient unit. These patients included moderate risk cardiac patients who were being observed (inpatient admission <24 hours) and cardiac patients who had to undergo an invasive procedure, such as catheterization, percutaneous coronary intervention (stents), or bypass. The low-severity group (e.g., those with no cardiac event) comprised patients who had the same cardiac symptoms and diagnostic tests performed at presentation to the ED but, after an evaluation, were discharged home without an inpatient hospitalization. 

### 2.3. Procedures

Patients were approached as close to the end of their visit as possible, which was anticipated through review of their medical chart and discussion with the medical team. This strategy helped to ensure that the majority of the entire health event had transpired prior to interview, which was deemed important considering our interest in how these constructs changed over the course of the event. While it would have been ideal to catch individuals very early in their visit, and assess their reactions in “real time,” practical and ethical considerations made this impossible.

Inclusion criteria for the participants were (1) adults ≥ 18 years old, (2) presenting with chest pain, chest pressure, shortness of breath, or syncope, (3) having a cardiac evaluation consisting of at least an electrocardiogram and cardiac enzyme tests, and (4) being an active smoker of ≥1 cigarette per day. Exclusion criteria were (1) presentation with drug or alcohol abuse, (2) presenting symptoms and complaints resulting from trauma; and (3) inability to participate in an interview (e.g., severe medical illness, cognitive insufficiency, insurmountable language barrier).

To reduce selection bias, at the time of interview the research assistants reinforced the concept that the study was observational, and participants did not have to want to change their behaviors to participate. Eligible and consenting patients signed a written informed consent. The semi-structured interview was recorded using a digital recorder and generally took less than 60 minutes. Participants were provided with a pamphlet on community-based smoking cessation services but were not otherwise compensated for participation. The interviewers, graduate clinical psychology students, were all trained to be mindful of the patient's state of mind and wellbeing, to halt the interview if the patient seemed unduly distressed, and to seek assistance from a clinician if it seemed warranted for any reason.

### 2.4. Materials and Measures

The items on the semi-structured interview consisted of qualitative, open-ended questions, as well as fixed answer (e.g., yes/no), and 7-point Likert-style questions. To reduce bias, the interviews started with the broad, open-ended questions before incorporating drill-down questions about specific constructs of interest, including perceived illness severity, event-related emotional reactions, and causal attributions. Reflective listening and summative statements were utilized to encourage elaboration in response to the open-ended questions before closed-ended and quantitative ratings were obtained. The semi-structured technique used fixed questions but allowed interviewers to ask unscripted, relevant follow-up questions for clarification.

#### 2.4.1. Perceived Illness Severity

Perceived illness severity was self-reported by the participant for three different time anchors: (1) when the participant first began to experience the symptoms that brought him/her to the hospital “symptom onset”, (2) when the participant decided to come to the hospital “hospital decision”, and (3) at the time of the research interview near the end of the visit “at interview”. After obtaining a general chronology and description of the illness, questions about perceived illness severity began with a qualitative question “I am interested in hearing about how serious you thought your problems were <insert time anchor>.” It ended with a quantitative rating of how serious he/she thought the problem was on a 7-point scale, where a 1 indicated “not at all severe” and 7 indicated “life threatening.” The process was repeated for each of the three time anchors. In addition to the three time-anchored ratings, average perceived illness severity and change in perceived illness severity from the “symptom onset” or “hospital decision,” whichever was highest, to “at interview” were calculated.

#### 2.4.2. Event-Related Emotions

Emotional reactions were measured using open-ended questions about the participant's emotional state during the same three time anchors (e.g., “At first, what kind of emotions or feelings did you experience with your physical symptoms?”). The particular emotion reported as being strongest by the participant in the qualitative question was then rated on a 7-point Likert scale for the strength of that emotion, where 1 indicated “not present” and 7 indicated “extremely strong.” For example, if the participant reported that his most pronounced emotion was fear, he then rated the strength of his fear on the 7-point scale. For quantitative analysis, a single variable was created that reflected the valence and intensity of the strongest emotional response. The Positive and Negative Affect Schedule (PANAS-X) [24] was used by two independent raters to code each self-expressed strongest emotion as positive or negative, and the Likert scales on which they were rated were recoded into a 15-point emotional intensity scale from −7 (strongly negative emotion) to +7 (strongly positive emotion), with no emotional response coded as zero. Any disagreement in positive versus negative valence by the two raters was adjudicated by a third rater to reach a final decision. Average emotional intensity ratings were calculated across the three time points, and change in emotional valence from the worst point to the time of interview was also calculated.

#### 2.4.3. Causal Attribution

Participants were first asked an open-ended question, “What do you think caused your health problem,” and were prodded with follow-up questioning to list any and all contributors. Data were coded to indicate whether smoking was mentioned spontaneously in this list of contributors. If the participant did not mention smoking spontaneously during the open-ended query process, he or she was asked directly, “Do you think your health problem could be related to your smoking?” and answers were coded as “yes,” “no,” or “maybe/unsure.” Finally, all participants were asked to rate how strong they believed that the link between smoking and their current health problem was using a 7-point scale, where 1 indicated “no link” and 7 indicated “extremely strong link.” Because causal attribution was conceptualized as being more stable, and early interviews found it awkward to ask about causal attribution repeatedly, it was assessed only once and anchored to the “current health problem” that brought the participant to the hospital rather than reassessed at each of the three time anchors, like perceived severity or emotional reactions.

### 2.5. Data Analysis

The mixed qualitative and quantitative design employed a concurrent triangulation approach [25], in which both qualitative and quantitative data were collected from the same participants and took on equal weight in our evaluation of results.

#### 2.5.1. Qualitative Data Synthesis

We used the framework approach [26] within Grounded Theory to identify the thematic structure, index the data, reorganize and distill the data by themes, and map the range and nature of the constructs observed. The interviewer summarized each individual interview, using detailed notes and audio recordings of the interviews for reference. When possible, the interviewer included key phrases and sentences to add depth to the concepts expressed by the participant. The individual theme summaries were organized by each of the three key constructs. Each participant's theme summary was blended into a master summary document by one of the authors (K.T.). When an individual participant's theme already existed in the master document, the individual's theme was added beneath the existing theme. When it did not already exist, a new theme was created. This was performed until we reached theme saturation, which was defined as five consecutive interviews for which no new themes were identified. Although all 50 subjects were interviewed, theme saturation, defined as five consecutive interviews for which no new themes were identified, occurred after the 35th interview. The final organization and distilling of content areas was performed on a consensus basis by the authors (E.B., K.T., B.B., and E.O.). Typical examples judged representative by two authors (E.B., K.T.) are provided in the Results. 

#### 2.5.2. Quantitative Analysis

All quantitative analyses were performed using PASW 17.0 (SPSS, Chicago, IL USA). Descriptive statistics, including measures of central tendency and variability, were calculated for the 7-point Likert-type ratings of the three constructs. Linear associations between the constructs (both within construct over time and across construct) were examined using Pearson correlation coefficients. We examined group differences in the constructs based on location of enrollment (ED versus inpatient), and we compared demographic variables between those patients who enrolled and those who did not. Independent samples *t*-tests and chi-square tests (including Fisher's Exact) were used to compare group differences in the variables of interest. 

## 3. Results

### 3.1. Participants

**Figure 1:**
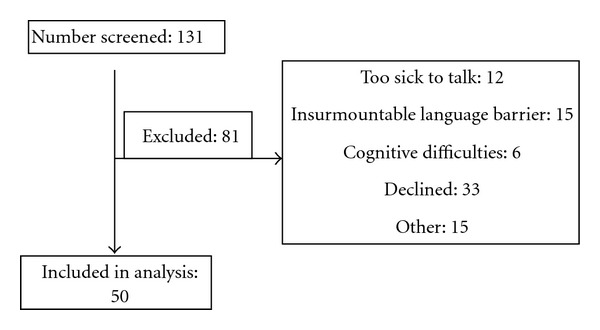
Screening and enrollment.

We screened 131 patients, and 50 participants (30 men and 20 women) completed a semi-structured interview (see Figure 1). The average age of participants was 50.6 (±12.4) years old, with a median age of 48.5, and ranged from 23 to 82 years old. The sample comprised 66% whites and 34% blacks, with 10.3% of these identifying themselves as Hispanic. The only significant difference between those enrolling and those not was in Hispanic ethnicity identification, such that Hispanic patients were less likely to enroll (*P* < 0.05). This may reflect the fact that some Hispanic individuals living in the catchment areas of both hospitals did not speak fluent English and could not complete the interview.

### 3.2. Qualitative Findings

#### 3.2.1. Perceived Severity

Less than half of participants (16/50, 32.0%) reported perceiving their symptoms as serious when the symptoms first started. Patients often anchored their severity ratings with reference to specific illnesses or conditions, like pneumonia, a heart attack, heartburn, or upper respiratory infection. Most patients indicated that they initially thought that they were experiencing a minor physical ailment, such as heartburn or anxiety, which may reflect the anomaly of such a health event, the lack of understanding of certain symptoms, or the denial that such an event could occur

* At first I thought that it was congestion, I have had pneumonia before and this felt similar, it was cold outside and I thought the pain was from the cold air. I should have known what the symptoms meant.* (age 59 male, ED)**

* I've been under tremendous stress and when the pain started, I was extremely angry. I did not think it was too bad. I thought it would go away when I got off the phone from arguing with my wife.* (age 54 male, ED)**

Not unexpectedly, by the time the participants decided to come to the hospital, many more of them perceived the condition as serious (41/50, 82.0%). For many, this change occurred because they reported that the symptoms had gotten worse or because they were instructed by others to come to the hospital. Of note was the particular importance for many participants of family members in making the decision to come to the hospital, suggesting that many individuals continued to experience some sort of denial about the potential seriousness of the situation

* I had a bite to eat and I got heartburn and it was not going nowhere … then the pain got a little worse and I threw up so I thought, something's wrong here, so I called the Emergency Room … my daughter-in-law said, “do you want to go to the hospital” and I said, “yeah, we better,” because my symptoms were getting worse. I've had two heart attacks in the past so I'd better not ignore it*. (age 62, female, inpatient)**

* I thought it was serious for a short time yesterday but after resting it went away, I raked in the back yard so my concern went away. My wife decided to take me to the doctor. After I went to the doctor, I was not concerned still, I would have gone home if the doctor hadn't said to come here*. (age 59, male, ED)**

At the time of interview, towards the end of the healthcare visit, the prevalence of participants perceiving their current condition as severe had reduced to 20 out of 50 (40.0%). Six thought that their current condition had decreased in severity, and 11 indicated that it was no longer serious at all (most common among those given a noncardiac diagnosis). Others were uncertain. However, as the participants looked back on the entire situation, 44 out of 50 thought that the condition was serious enough to warrant coming to the hospital.

* It's very serious and it's up to me to change it… to make some changes to make sure it does not happen again*. (age 46, male, inpatient)**

* I figure it's not that serious anymore since the pain is going away on its own*. (age 47, female, ED)**

Severity categories are shown in appendix.

#### 3.2.2. Emotions Reported

Participants expressed a wide range of positive and negative emotions across the three time anchors, providing a total of 40 unique adjectives or descriptors (see Table 3). Many subjects provided multiple adjectives to describe each time point. Emotional valence was generally negative at symptom onset (78 negative and two positive adjectives expressed overall) and upon hospital arrival (67 negative expressions and 6 positive) but grew notably more mixed toward the end of the healthcare encounter (48 total expressions of negative emotions and 23 positive ones). This pattern validated that we reached individuals toward the end of their visit, once they had been assessed and treated and/or their symptoms had improved. Positive emotions were more commonly expressed by those who had completed treatment or had been told that their condition was minor in nature. Anxiety was still the most commonly expressed emotion (*n* = 16), but it was followed closely by happiness (*n* = 11). It seems reasonable to expect that since all these patients were still in the hospital, that negative emotions would continue to predominate, and that discharge (or even just news of impending discharge from hospital) would then precipitate more positive emotions.

* Frustrated because here we go with these palpitations, because they're annoying… I was mad, you do not want to feel like this at 1 o'clock in the morning*. (symptom onset) (age 48, female, ED)**

* I was scared… I do not know what of… my mom died, my father died, my daughter died of congestive heart failure… I was feeling very sad*. (symptom onset) (age 62, female, inpatient)**

* I was anxious… just wanna know what's wrong…* (at hospital decision) (age 45, female, ED)**

* I do not know what they're going to find in my blood, just the worrying about that. Want to know if it's treatable and just move on*. (at interview) (age 45, female, ED)**

* I'm feeling quite nice and happy because it's all over*. (at interview) (age 54, male, ED)**

One notable observation was the difficulty that some participants seemed to have in finding words to describe their emotions. Many described events, symptoms, and thoughts, rather than emotions, had to be prompted to further describe an emotion or feeling. Two were never able to describe an emotion or denied that they had felt anything across all three time points

* I wasn't feeling any emotions. This kind of thing has happened before. I just did not want to go to the hospital, I wanted to stay home*. (symptom onset) (age 47, female, ED)**

In total, there were 15 cases of denied or lack of emotion at the time of the event, 10 at the time of presentation to the hospital, and 4 at the time of interview. The increase in ability to identify or acknowledge emotion was notable, perhaps suggesting that some individual simply had difficulty remembering emotions during such a tumultuous experience, a not-unreasonable form of memory bias.

#### 3.2.3. Causal Attribution

Participants mentioned a plethora of factors they believed to have contributed to their current health problem in response to the open-ended question. Some participants readily recognized the strong link between smoking and their current health problem

* Strong link [between smoking and health problem]. The doctor just told me, if I do not stop smoking cigarettes, he said I'm bound for a heart attack. I do not want that. I'm not ready to say, “I'm ready to die” or “I'm just giving up,” but cigarettes is the hardest thing I've ever had to kick. I do not drink, I do not smoke weed but I do smoke cigarettes. I've been doing that since I was 17 and I'm 45 now. I've ignored it for so long but I cannot ignore it anymore*. (age 45, female, ED)**

In contrast, many denied that smoking was contributory, even when asked directly. Of note was the number of patients who could cite a particular individual who lived healthfully until old age despite smoking, a rare co-occurrence of events and a classic example of “illusory correlation.”

* I been smoking for 56 years, my lungs are all black, but I do not smoke now unless I get upset… so I do not think it's related to my health problem*. (age 71, female, ED)**

* I just do not see a connection. I knew someone who was up in age and smoked since she was a little girl and never had a problem with her health. Never*. (age 62, female, inpatient)**

One odd finding was the couple of patients who indicated that smoking could not be causal because they had been doing it for so long without having any prior issues, for example, “No, it's not the smoking because I've been smoking my entire life and never felt anything like this before” (age 55, female, inpatient.). This seems to suggest that these patients were under the unfortunate impression that smoking-related health problems would occur quickly, or not at all.

Participants clearly viewed their condition as multicausal. They mentioned other deleterious health behaviors (e.g., alcohol and drug use, poor weight control, lack of exercise) noncompliance with physician recommendations, family history, and psychological issues.

* Lack of exercise. Too much stress in my life. Stress would probably be the biggest thing, owning my own business. I think that's the root of all evil*. (age 54, male, ED)**

* I know that smoking is one of [the causes]. Another inherited trait… my mother, my dad, my sister, my brother, we all have high blood pressure. I do not think that's something I could escape… the most important thing in my family. Also stress*. (age 45, female, ED)**

Of particular note was the number of people mentioning causal attributes external to their own control, such as genetics/family history and stress. We were particularly surprised at the number of people relating psychological issues with health conditions, leading us to hypothesize about whether the mass media's recent growing interest in the effects of stress and depression on physiology and health was leading some people to excessively externalize responsibility for their health problems.

### 3.3. Quantitative Findings

#### 3.3.1. Descriptive Statistics

**Figure 2:**
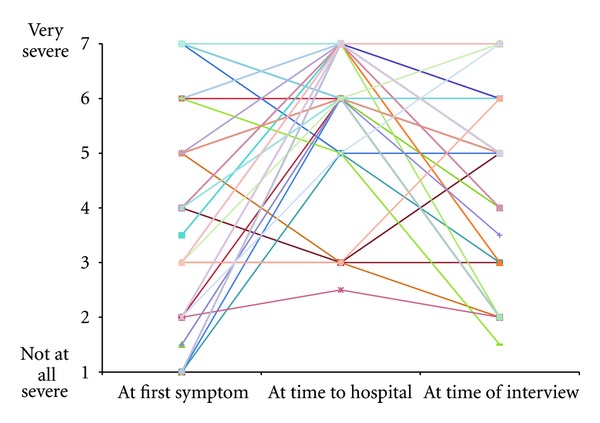
Patterns of severity ratings for individual participants over time.

**Figure 3:**
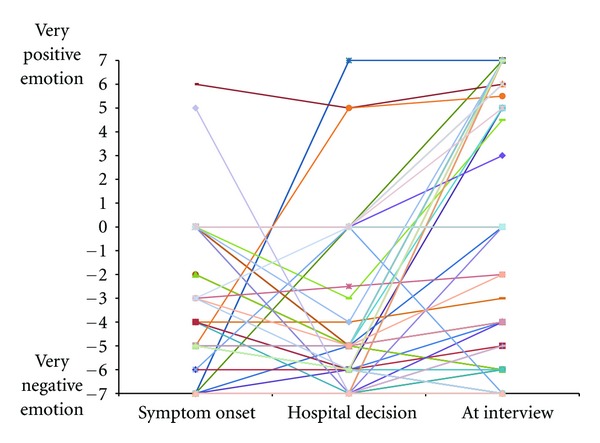
Patterns of emotional valence for individual participants over time.

Descriptive statistics are shown in Table 1. Figures 2 and 3 depict the perceived severity and emotional reaction ratings over the three time anchors. The pattern for perceived illness severity suggested that, on average, perceived severity fell in the middle of the 7-point scale at symptom onset (4.00 ± 2.20), peaked at time of deciding to go to the hospital (6.17 ± 1.27), and decreased from their peak by the time of interview toward the end of their visit (4.94 ± 1.82).

The emotion valence and intensity ratings (on a scale from −7 to +7) suggested strong negative emotions at symptom onset (−3.82 ± 3.29), which persisted or increased through hospital decision (−3.99 ± 3.83), followed by less negative and more positive emotions by the time of interview (−0.29 ± 5.76).

Of 50 participants, 26 (52%) spontaneously mentioned smoking as a causal factor for their health problem without being prompted. Of those who had to be prompted (those who did not spontaneously report it and who were asked “Do you think your health problem could be related to smoking,” only three out of 24 (13%) indicated “yes,” leaving 21 (42%) who denied a link between smoking and their symptoms. All participants were asked to provide the 7-point rating, resulting in an average connection between smoking and the current health problem of 4.79 ± 2.09.

#### 3.3.2. Correlations

Table 2 presents the correlation coefficients for perceived illness severity and event-related emotional response, both within and between the three time points. For both perceived illness severity and event-related emotional response, the strongest correlation was between the assessment corresponding with the decision to come to the hospital and the time of the interview (i.e., towards the end of their stay). Between constructs, the correlations were generally nonsignificant. The notable exception was the correlation between the perceived illness severity at the onset of symptoms and event-related negative emotional response at onset of symptoms (*r* = − .43) and at time of interview (*r* = −.28). The more severe the onset of symptoms, the more negative the emotional response across the healthcare encounter.

Smoking-related casual attributions did not correlate significantly with the three indicators of perceived illness severity: symptom onset, *r* = .05, *P* = .71; hospital decision, *r* = .19, *P* = .18; at interview, *r* = .27, *P* = .06. Similarly, causal attributions did not correlate significantly with the three event-related emotional response indicators: symptom onset, *r* = −.11, *P* = .43; hospital decision, *r* = −.02, *P* = .87; at interview, *r* = .19, *P* = .20.

#### 3.3.3. Comparison of Participants in the ED versus Inpatient Units

Perceived event severity, emotional reactions, and smoking-related causal attributions were not statistically different between inpatients and ED patients (see Table 1, all *ps* > .05). 

## 4. Discussion

The mechanisms of action whereby a health emergency inspires both short- and long-term behavior change have been poorly defined [27]. Our study gathered data to help operationalize three constructs related to experiencing suspected cardiac symptoms. In addition to providing rich information on adjectives and descriptors that can be integrated into item construction, the results hold three important implications for measurement and hypothesis generation. First, measures will likely require multiple items to cover the breadth and multidimensionality associated with each construct. Second, both cognitive and affective constructs should be considered. Third, repeated assessment of these constructs over serial time anchors, rather than aggregated or global ratings, should be obtained.

The perceived severity of an acute health event, while having strong roots in constructs from the Health Belief Model [13], is difficult to measure and is fraught with unanswered questions. Constructing a scale requires first-hand information about adjectives patients use to reflect severity (see appendix). When asked in an open-ended fashion, patients experiencing suspected cardiac symptoms were readily capable of describing their perceptions of how serious their health problem was at symptom onset, decision to present to the hospital, and at the time of the interview, which occurred towards the end of their healthcare encounter. While many subjects used traditional adjectives like “serious” or “minor,” others referenced specific illnesses they thought reflected levels of severity, like “I thought I had pneumonia” or “I thought it was just heartburn.” Future scale construction may need to include both traditional adjectives and illness-specific descriptors to fully assess perceptions of severity.

 Several patterns reinforce the importance of maintaining multiple time anchors. The narratives, reinforced by changes in the 7-point ratings over the three time anchors, suggested that profiles varied considerably across individuals. For example, one common pattern was reflected by low severity at symptom onset, a spike at initiation of visit to the ED, and a subsequent decrease towards the end of the healthcare encounter (i.e., low-high-low). However, as Figures 2 and 3 depict, other patterns were reported, such as high perceived severity at symptom onset that remained elevated throughout the other two time anchors (i.e., high-high-high). This variability in ratings over time implies that one single aggregate rating would have been incapable of representing the experience. The correlation coefficients across time anchors reinforced this interpretation. For example, severity at symptom onset had virtually no relation with severity at hospital presentation. Although there is no universal method to determine what these time anchors should be, logically, they should probably span across the event. This measurement strategy, though complex and characterized by limitations, such as relying on retrospective recall, is nevertheless worthy of consideration. We currently know nothing about whether health behavior change is more likely in patients with a particular profile (i.e., low-high-low versus high-high-high), or if it is simply an average of the various time points, or even the maximum peak severity rating regardless of when it occurs. Serial assessments are required to shed light on the nuances of these relations. Future research should ensure that all patients are measured at the same point in their care, or measure at multiple time points to ensure reliability of results.

Although the general assumption in the health behavior change literature is that negative effect promotes relapse and retards behavior change, negative emotional reactions to a sentinel health event may actually be a motivator of change. Some novel research has demonstrated that anxiety and a sense of “looming vulnerability” can lead to an increase in smoking cessation attempts [28]. In another study, level of worry correlated positively with quit attempts, particularly in those with high self-efficacy and strong beliefs in the value of quitting [29]. The emotional response to acute illnesses can be complex and requires further exposition. In our sample, many of the same patterns and conclusions that applied to perceived illness severity could be applied to event-related emotional reactions. The adjectives used to describe emotional reactions were quite varied and argued for multi-item assessment of both positive and negative emotions (see appendix). The narratives clearly supported that the emotional reactions changed as the event unfolded. For example, positive emotions, like happiness or relief, were very rare at symptom onset and hospital decision, but occurred relatively frequently toward the end of the visit, perhaps as a response of decreased symptoms, medical treatment, and physician reassurance. Correlations between ratings at different time points were poor or modest, also reinforcing the importance of serial assessments. Finally, one notable observation was the difficulty some individuals had in identifying an emotional reaction. In response to the open-ended question about emotions or feelings they may have experienced, some individuals provided cognitive reactions or symptoms, rather than emotions. Several reported experiencing no emotion at all. In such patients, this response did not seem to be accounted for by low illness severity since several had serious medical illnesses for which they were hospitalized. If a negative emotional reaction to a sentinel event inspires change, a complete absence of an emotional reaction may be an important moderator of the health event behavior change relationship although its nature is yet unclear and warrants further study.

We expected perceived illness severity and negative event-related emotional reactions to be higher among admitted patients, when compared to ED patients who were discharged home, since admission status is generally viewed as a global indicator of severity. However, this expectation was not confirmed, as there were no significant differences in any of the measures when comparing ED patients and inpatients. This is difficult to interpret though it seems unlikely to be due to a ceiling or floor effect, such that all subjects rated their illnesses as either very serious or minor. Standard deviations of the perceived severity ratings at each time anchor reflected considerable variability between subjects (see Table 1), and, while the distributions were not normal, they were not dramatically skewed. The lack of differences between ED patients discharged home and inpatients further supports the importance of investigating these perceptions and emotional reactions and how they relate to actual illness severity. There may not be a strong tie between personal reactions to events and their actual severity, which could, in part, explain why some people continue to maintain unhealthy behaviors in the face of medical problems that are considered life threatening by healthcare professionals.

Finally, even in the face of decades of massive public education campaigns, many individuals in our study remained unaware of that the role smoking may play in their cardiac health, a pattern that is consistent with previous research [8]. Nearly half did not mention smoking as a potential causal factor when queried. Such findings emphasize the importance of the “teachable moment” in such settings like the ED; providing these patients with education about the possible relation of their illness to their tobacco use may increase cessation attempts, as reported in previous studies [7, 8]. Moreover, our results confirmed that causal attribution is multidimensional and complex. Most individuals readily identified multiple reasons for their health problem. This multi-dimensionality may have important implications for measurement and testing hypotheses in future studies since it suggests that assessing only smoking-related attributions independent of other causal dimensions may yield a limited picture.

There are a number of limitations that must be noted regarding the present study. First, it was primarily exploratory and not designed to test how the constructs actually related to behavior change; that objective is for future studies, which can be informed by the results of this study. Second, evaluating a person's thought processes over several time points would best be evaluated using a prospective format at multiple time points, in order to eliminate risk of recall bias. However, interviewing a patient when their symptoms first start, or when they first arrive to the hospital, is neither feasible nor ethically acceptable. Nevertheless, relying on an individual's later recollection of how they felt at the start of an acute health event can possibly be biased by the events that occurred since that time. Third, because this was a pilot study designed to help build future standardized instruments, each quantitative measurement was represented by a single item. However, our own results strongly suggested that multi-item scales for each construct are critical. Consequently, the relations between our single-item constructs may misrepresent the true relations and should be interpreted with caution.

## 5. Conclusions

The relation between an acute health event and smoking cessation appears to be very complex. It probably depends not only on cognitive perceptions, like illness severity and causal attribution, but also on emotional reactions. Moreover, it may act in the context of other chronic life events and back-ground constructs, such as previous events, baseline motivation, self-efficacy, and social factors, like prosmoking relationships or environments. Our results suggest that assessment of event-related perceptions and emotional reactions will likely need to be multi-item, multidimensional, and rated serially over the event chronology. Measurement of causal attribution must likewise be multi-dimensional, as patients rarely attribute their illness to a single cause, like smoking.

## Figures and Tables

**Table 1:** Descriptive statistics of constructs.

Variable name	ED with discharge (*n* = 22)		Inpatient unit (*n* = 28)		All (*n* = 50)	
	Mean	S.D.	Mean	S.D.	Mean	S.D.
Perceived event severity						
(1, not at all serious to 7, extremely serious)						
Symptom onset	3.68	2.14	4.25	2.27	4.00	2.20
Hospital decision	6.09	1.19	6.23	1.34	6.17	1.27
At interview	4.81	1.91	5.04	1.77	4.94	1.82
Average	4.87	1.01	5.13	1.36	5.03	1.74
Delta	1.28	1.95	1.32	1.62	1.28	1.74
Even-related emotional intensity						
(−7, very negative to +7, very positive)						
Symptom onset	−4.27	2.57	−3.46	3.77	−3.82	3.29
Hospital decision	−4.55	3.10	−3.55	4.33	−3.99	3.83
At interview	−1.39	5.33	0.66	6.03	−0.29	5.76
Average	−3.28	3.08	−2.05	3.25	−2.74	3.23
Delta	3.93	4.52	5.50	6.20	4.78	5.48
Causal attribution						
(1, no relation to 7, very strong relation)	4.50	1.79	5.02	2.30	4.79	2.09

Note: no significant differences were found between discharged and admitted patients on these measures.

**Table 2:** Correlation coefficients for perceived event severity over the three time anchors.

	Perceived severity at symptom onset	Perceived severity at presentation to hospital	Perceived severity at time of interview	Emotional valence at symptom onset	Emotional valence at presentation to hospital	Emotional valence at time of interview
Perceived severity at symptom onset	1.00					
Perceived severity at presentation to hospital	.09	1.00				
Perceived severity at time of interview	.14	.39^∗∗^	1.00			
Emotional valence at symptom onset	−.43^∗∗^	−.23	−.28^∗^	1.00		
Emotional valence at presentation to hospital	.07	−.09	−.19	.24	1.00	
Emotional valence at time of interview	.06	−.17	−.15	.18	.48^∗∗^	1.00

^∗^ *P* < 0.05 level (2-tailed), ^∗∗^ *P* < 0.01 level (2-tailed).

**Table 3:** Adjectives used to describe emotions by patients with suspected cardiac symptoms as a metric of commonality of experienced emotion.

At first	At time to hospital	At time of interview
Fear/scared (21) Anxiety/nervous/ worried (21) Anger (8) Aggravated/frustrated/ annoyed (2) Depressed/sad (6) Concerned (5) Stress (4) Embarrassed (2) Calm (1) Confused (1) Disoriented (1) Dread (1) Extremely nervous (1) Relaxed (1) Upset (1) No emotion/denial (15)	Fear/scared (28) Anxiety/nervous/ worried (9) Anger (6) Aggravated/frustrated/annoyed (2) Depressed/sad (4) Concerned (2) Stress (1) Relief (2) All right (1) Bad (1) Calm (1) Dread (1) Embarrassed (1) Grateful (1) Hopeful (1) Shock (1) Sorry (1) Upset (1) No emotions/denial (10)	Anxious/nervous/ worried (16) Happy (11) Fear/scared (10) Relief (8) Depressed/sad (6) Aggravated/frustrated/annoyed (7) Stressed (4) Relaxed (3) Feels good (2) Calm (2) Glad (2) Fine (2) Excited (1) Peaceful (1) Grateful (1) Dread (1) Angry (4) Embarrassed (1) Concerned (2) Lonely (1) Upset (1) Hurt (1) Disgust (1) Disappointed to be in hospital (1) Unsure (1) Funny (1) Safer (1) No emotions/denial (4)

Note: Each participant may have expressed more than one emotion.

## Appendix

*Adjectives Used to Describe Severity and Emotional Reaction to Suspected Cardiac Symptoms*

(1) Serious

Very/real/pretty serious
 Life threatening
 Thought it was a heart attack or stroke
 Thought (s)he was going to die
 Scared (of dying)
 Bad
 Knew was serious from previous experience
 Knew something was wrong
 Serious enough to call rescue
 Serious but not life threatening
 Concerned

(2) Not Serious

 Not serious “at all,” “not too serious,” “mild severity”
 Thought it was indigestion/heartburn/gas
 Thought was a more minor condition (virus pulled muscle)
 At first pain not intense so not concerned
 Thought it would go away after leaving stressful situation
 Thought it was an asthma attack
 Did not think much about it
  “I did not”
 Would have gone home if MD had not told him to come to ED
 Feels okay about it
  “All right”
Not worried now
 Fine

(3) Decreased Severity (over the Course of the Event)

Not serious since know now it is not the heart
Not serious because symptoms are disappearing
Not serious because being cared for
Concerned but not as worried

(4) Increased Severity (over the Course of the Event)

More serious than originally thought
More serious because symptoms got worse

(5) Uncertain about the Severity

Do not know
Waiting for information from health care professional
Waiting for outcome of procedure.
